# Disordered eating behaviors in university students in Hanoi, Vietnam

**DOI:** 10.1186/s40337-015-0054-2

**Published:** 2015-04-01

**Authors:** Nayeong Ko, Duong Minh Tam, Nguyen Kim Viet, Peter Scheib, Michael Wirsching, Almut Zeeck

**Affiliations:** Department of Psychosomatic Medicine and Psychotherapy, Freiburg University Medical Center, Hauptstrasse 8, Freiburg, 79104 Germany; National Institute of Mental Health, Hanoi Medical University, 78 Giai Phong Street, Dong Da District, Hanoi, Vietnam

**Keywords:** SCOFF questionnaire, Eating disorder Inventory 2, Body mass, Eating disorders, Female students, Vietnam

## Abstract

**Background:**

The aim of the study was to examine disordered eating behaviors in university students in Vietnam.

**Methods:**

A total of 244 female university students participated, and 203 data could be analyzed. The Body Mass Index, the SCOFF screening questionnaire and the Eating Disorder Inventory 2 were used to explore disordered eating behaviors.

**Results:**

45.3% of the participants were underweight, 53.2% were normal weight and 1.5% were overweight. 48.8% of students reported two or more yes-responses on the SCOFF screening questionnaire which indicates a high possibility of having eating disorder symptoms. The mean score for underweight subjects (M = 14.79, SD = 6.81) indicated a lower level on the drive for thinness scale of the EDI-2 compared to normal weight subjects (M = 24.65, SD = 6.86) and overweight subjects (M = 31.33, SD = 6.66). Additionally, underweight subjects (M = 27.24, SD = 7.57) were less dissatisfied with their body than normal weight subjects (M = 35.94, SD = 8.67) and overweight subjects (M = 43.33, SD = 11.24). A significant positive correlation appeared between the BMI and the EDI-2. The SCOFF questionnaire showed a statistically significant negative correlation with the BMI and the EDI-2.

**Conclusions:**

Despite some limitations the current study shows a tendency in young females in urban Vietnam to be underweight and to develop disordered eating symptoms such as drive for thinness and body dissatisfaction. However, more studies using the SCOFF and the EDI-2 would be needed to verify these findings.

## Background

Eating disorders are one of the most relevant mental disorders not only in Western countries but also in non-Western countries. Recently, reviews have shown that the prevalence of eating disorders in non-Western countries is not consistently lower compared to the prevalence in Western countries [1]. Eating disorders do occur all around the world [2]. Many important factors influence the increase in eating disorders worldwide. Some experts suggested that globalization and internationalization have brought the beauty ideal and pursuit of thinness, as well as the value of beauty and social norms of Western cultures to non-Western cultures, societies, and people, which, in turn, may lead to an increase in the occurrence of eating disorders [3-5]. Furthermore, some cross-cultural studies reported on an increase of eating disorders in non-Western societies, due especially to economic development, the exposure to Western culture, the higher socioeconomic status of individuals and so forth [6-8]. Although these studies evinced that eating disorders are increasing in many non-Western countries, there is inconsistency in the methods used and some reported results. The question regarding the factors which are most likely to cause the development of eating disorders as well as the difficulties in measuring the current prevalence of eating disorders in non-Western countries are issues that must be addressed. More study results are needed, especially from non-Western countries regarding prevalence rates and the factors associated with eating disorders in those countries.

One challenge in evaluating the results of our own study in Vietnam was the lack of studies that examine eating disorders in this part of the world and are published in international journals. Tsai [9] reported in his review on the prevalence of eating disorders in Asian countries that no studies on Vietnam existed at that time. Very few studies on body image or obesity have included the Vietnamese as participants, especially since their economic reform. Vietnam is one of the fastest developing Asian countries and is quickly changing culturally and socially. The economic growth in Vietnam has rapidly increased since the economic reform program that was termed Doi Moi [10]. Lifestyles and dietary patterns of the Vietnamese population have been changing during this economic development [11]. Additionally, the nutrition conditions have changed and improved through the national nutrition policies from 1995 to 2000 [10].

After the economic reform and the improvement of nutrition policies, some national surveys were conducted and report data regarding the prevalence of obesity and thinness in the Vietnamese population. For example, the results described by Tuan et al. [10] from the data of the Living Standard Survey in 1992 and the National Health Survey in 2002 showed that the prevalence of obesity and being overweight generally increased more in urban areas than in rural areas, and the prevalence of being underweight decreased in adults from 1992 to 2002 in Vietnam. It has also been reported from the data of the National Nutrition Survey in 2000 and the National Adult Obesity Survey in 2005 that the prevalence of being overweight or obese was higher in 2005 as compared to 2000 in both rural and urban areas, with the highest prevalence in urban areas [11]. The prevalence of being underweight was lower in 2005 than in 2000 in both areas, but higher in rural areas compared to urban areas in both years [11]. Additionally, the higher rates of being overweight in the Vietnamese people were positively linked to socio-economic status indices [12]. The prevalence of being overweight or obese in Vietnam is lower as compared to the findings in other Western countries [13]. However, the prevalence of being overweight and obese has rapidly increased in Vietnam with the economic development and the changing lifestyle which has been influenced by Western cultures.

Unfortunately, there are no further studies published in English language journals on the prevalence of eating disorders in Vietnam. From the few results showing the prevalence of being overweight and obese, it could be assumed that some symptoms of bulimia nervosa, anorexia nervosa, disordered eating behaviors, or body dissatisfaction might also appear in Vietnam currently. In particular, disordered eating behaviors and eating disorders have often been observed in association with economic development, rising incomes, and economic prosperity with females being more susceptible than males [8,14]. It can be assumed that eating disorder behaviors could be observed in young Vietnamese female students who are easily exposed to these diverse influencing factors that are currently discussed in the research context.

Hence, the aim of this study was to look for disordered eating behaviors in female university students in Vietnam. Given the extreme changes in society and lifestyle as well as the rapid economic development, one would expect to find disordered eating behaviors in female university students in Vietnam. In the current study the SCOFF questionnaire [15] was used to screen for disordered eating patterns, while the Eating Disorder Inventory 2 [16] was used to examine disordered eating behaviors in more detail.

## Methods

### Participants

A Vietnamese researcher visited a number of lectures at the Hanoi Medical University and briefly introduced the current study. The students voluntarily took approximately 15–20 minutes to complete the questionnaires after their lectures. A total of 244 female students from the Hanoi Medical University in Vietnam participated in the study. 41 incomplete questionnaires were excluded from data analysis. Therefore, the response rate was 83.2% and a total of 203 female student questionnaires (age range from 17 to 31) were used for the statistical analysis.

### Procedure

The questionnaires of the current study were translated from English into Vietnamese by one bilingual psychiatrist, the translated questionnaires were then back-translated by another bilingual psychiatrist. After discussion with one further Vietnamese psychiatrist, the translated questionnaires were finally modified for the current study.

### Measures

#### Body mass index

Self-reported height and weight were used to calculate the Body Mass Index (BMI). The BMI was calculated by dividing body weight in kilograms by the square of height in meters. The following classification was used: BMI ≥ 30.00 indicates obesity, BMI ≥ 25.00 overweight, BMI = 18.50 – 24.99 defines the normal range, BMI lower than 18.50 is considered as underweight [17]. There is also a recommendation how to classify the BMI in the Asian population by the WHO [17]: A BMI ≥ 27.50 indicates a higher risk for obesity, a BMI = 23.00 – 27.50 an increased risk of being overweight, a BMI = 18.50 – 23.00 defines the normal range, and a BMI of less than 18.50 is considered as underweight.

#### SCOFF questionnaire

The SCOFF questionnaire is an eating disorder screening questionnaire developed by Morgan et al. [15], which contains 5 short questions regarding key aspects of eating disorders such as vomiting, concerns about losing control over how much one eats, weight loss, feeling fat and whether food dominates life. The SCOFF questions read as follows:

1. Do you make yourself SICK because you feel uncomfortably full?
2. Do you worry that you have lost CONTROL over how much you eat?
3. Have you recently lost more than ONE stone in a 3-month period?
4. Do you believe yourself to be FAT when others say you are too thin?
5. Would you say that FOOD dominates your life?

These questions can be answered by ‘yes’ or ‘no’. Having two or more ‘yes’ responses on the SCOFF questionnaire indicates that the participant could have a high possibility of having anorexia nervosa or bulimia nervosa [15]. Some results from previous studies showed good reliability, effectiveness and a convenient administration of the SCOFF questionnaire [18,19]. Cronbach’s alpha of the SCOFF - score was 0.28 in the current study.

#### Eating disorder inventory 2

The Eating Disorder Inventory 2 (EDI-2) is a self-report measure with 91 items and 11 subscales developed by Garner [16]. The subscales are: drive for thinness, bulimia, body dissatisfaction, ineffectiveness, perfectionism, interpersonal distrust, interoceptive awareness, maturity fears, asceticism, impulse regulation, and social insecurity. Items are rated on a scale from 1 (never) to 6 (always). The EDI-2 has good reliability and validity values and is one of the most-used self-report measures for the assessment and screening of eating disorders. To examine disordered eating behaviors, three subscales were used in the current study. These consists of 23 items and include drive for thinness (DT), bulimia (B) and body dissatisfaction (BD). Cronbach’s alpha of the EDI-2 in the current study was 0.85.

### Data analysis

All variables defined in the study were analyzed by SPSS version 15.0 in English. The means and the standard deviations of age, Body Mass Index and other variables were calculated, as well as the reliabilities and the correlations between each item of the SCOFF. The three subscales of the EDI-2 were also analyzed. Analysis of variance (ANOVA) was performed to examine differences between groups.

## Results

Table 1 shows means, standard deviations and classification of BMI and age. The mean age of the participants was 18.80 years (SD = 1.47), and the BMI was 19.04 (SD = 1.90). The percentage of participants within the normal range of the BMI was 53.2%. The percentage of those who were underweight was 45.3%, and 1.5% of the participants were classified as overweight by the BMI classification of the WHO. According to the BMI classification for Asian populations, the result showed: 0.5% as obese, 3.4% as overweight, 50.7% as within the normal range, and 45.3% as underweight (see Table 1).

**Table 1:** **Descriptive statistics of the BMI**

***N = 203***	**Min**	**Max**	**M**	**SD**
Age	17.00	31.00	18.80	1.47
Body Mass Index (BMI)	14.84	28.30	19.04	1.90
*Classification*	BMI cut-off for worldwide*		BMI cut-off for Asian population**	
	N	Percentage	N	Percentage
Underweight	92	45.3%	92	45.3%
Normal weight	108	53.2%	103	50.7%
Overweight	3	1.5%	7	3.4%
Obese	0.0%	0.0%	1	0.5%

Note: * ≥30.00 = obesity, ≥25.00 = overweight, 18.50-24.99 = normal weight, ≤18.50 = underweight.

** ≥27.50 = higher high risk for obese, 23.00–27.50 = risk for overweight, 18.50–23.00 =  normal range, ≤18.50 = underweight (WHO [17]).

Table 2 shows the descriptive results for the EDI-2. The mean score of the EDI-2 was 64.44 (SD = 17.09), the mean of the subscale drive for thinness was 20.28 (SD = 8.48), the mean of the subscale bulimia was 12.05 (SD = 4.14) and the mean of the subscale body dissatisfaction was 32.11 (SD = 9.39).

**Table 2:** **Descriptive statistics of the EDI-2**

***N = 203***	**Min**	**Max**	**M**	**SD**
Eating Disorder Inventory 2 (EDI-2)	35.00	115.00	64.44	17.09
*Subscales of the EDI-2*				
Drive for Thinness (DT)	7.00	39.00	20.28	8.48
Bulimia (B)	7.00	29.00	12.05	4.14
Body Dissatisfaction (BD)	14.00	54.00	32.11	9.39

Regarding the SCOFF questionnaire, 48.8% of the participants reported two or more yes-responses (See Table 3). 101 of the 203 participants answered ‘yes’ to the SCOFF question ‘Do you worry that you have lost CONTROL over how much you eat?’, while only 3 participants answered ‘yes’ to the question ‘Have you recently lost more than ONE stone in a 3-month period?’ (see Table 3).

**Table 3:** **Descriptive statistics of the SCOFF**

***Item No. of the SCOFF***	***N*** **of yes-frequency (total** ***N*** **= 203)**	**Percentage**
1. make yourself sick (vomit)	40	19.70%
2. lost control how much you eat	101	49.80%
3. recently lost more than one stone	3	1.50%
4. believe yourself to be fat	84	41.4%
5. food dominates your life	70	34.5%
*SCOFF score*	*N*	Percentage
0-yes-response	46	22.70%
1-yes-response	58	28.60%
2-yes-responses	60	29.60%
3-yes-responses	36	17.70%
4-yes-responses	3	1.50%
5-yes-responses	0	0.00%

The correlations among measures are presented in Table 4. Data were inspected for normality and some data were not normally distributed and thereby the Spearman rank correlation statistic was used. The results showed that there was a significant positive correlation between the BMI and the EDI-2 (rho = 0.67, p < 0.0001). The SCOFF showed a negative statistically significant correlation with the BMI (rho = −0.15, p < 0.0383). The correlation between the SCOFF and the EDI-2 showed a significant negative association (rho = −0.29, p < 0.0001).

**Table 4:** **Spearman’s rho of the BMI, the SCOFF and the EDI-2**

***N*** **= 203**	**BMI**	**SCOFF**	**SCOFF1**	**SCOFF2**	**SCOFF3**	**SCOFF4**	**SCOFF5**	**EDI-2**	**EDI-2-DT**	**EDI-2-B**	**EDI-2-BD**
BMI	1										
SCOFF	-.15*	1									
SCOFF1	.05	.40*	1								
SCOFF2	-.15*	.71*	.10	1							
SCOFF3	-.07	.10	-.06	.04	1						
SCOFF4	-.18*	.50*	-.04	.08	.06	1					
SCOFF5	.01	.62*	.03	.34*	-.09	.04	1				
EDI-2	.67*	-.29*	-.06	-.24*	.00	-.30*	-.02	1			
EDI-2-DT	.69*	-.32*	-.07	-.30*	.01	-.27*	-.03	.86*	1		
EDI-2-B	-.09	-.19*	-.22*	-.11	.02	-.09	-.04	.26*	.02	1	
EDI-2-BD	.61*	-.17*	.02	-.15*	-.01	-.22*	.01	.87*	.61*	.06	1

Note: BMI = Body Mass Index, SCOFF = total 5 items of the SCOFF, SCOFF1 = item No.1 of the SCOFF, SCOFF2 = item No.2 of the SCOFF, SCOFF3 = item No. 3 of the SCOFF, SCOFF4 = item No.4 of the SCOFF, SCOFF5 = item No.5 of the SCOFF, EDI-2 = Eating Disorder Inventory-2, EDI-2-DT = Drive for Thinness subscale of the EDI-2, EDI-2-B = Bulimia subscale of the EDI-2, EDI-2-BD = Body dissatisfaction subscale of the EDI-2.

*Correlation is significant at the 0.05 level (2-tailed).

Analysis of variance (ANOVA) showed a significant difference between the drive for thinness score and the body dissatisfaction score of the EDI-2 in the 3 categories of the BMI classification, F (2,200) = 55.624 (p < .001), F (2,200) = 30.718 (p < .001), respectively. Furthermore, post hoc comparisons using the Tukey HSD test indicated that the mean score of the drive for thinness scale in the group being underweight (M = 14.79, SD = 6.81) was significantly different compared to the normal weight group (M = 24.65, SD = 6.86) and the group with overweight (M = 31.33, SD = 6.66). Additionally, the mean of underweight individuals (M = 27.24, SD = 7.57) was significantly different compared to the mean of normal weight individuals (M = 35.94, SD = 8.67) and overweight individuals (M = 43.33, SD = 11.24) on the EDI-2 scale body dissatisfaction.

In addition, the total SCOFF score in the SCOFF negative group and the EDI-2 showed a significant negative correlation (rho = −0.21, p < 0.0335) (see Table 5).

**Table 5:** **Spearman’s rho of the BMI, the SCOFF positive and negative groups and the EDI-2**

	**SCOFF positive group**	**SCOFF negative group**
	***N*** **= 99**	***N*** **= 104**
	**SCOFF**	**SCOFF**
BMI	.13	-.09
EDI-2	-.01	-.21*
EDI-2-DT	.02	-.18
EDI-2-B	-.14	-.03
EDI-2-BD	.03	-.15

Note: SCOFF positive group = more than 2-yes responses; SCOFF negative group = less than 1-yes response.

*Correlation is significant at the 0.05 level (2-tailed).

## Discussion

### Prevalence of underweight and overweight

Results show that 45.3% of the participating female university students in Vietnam were underweight. The previous national surveys from 1992 and 2002 reported that the percentage of underweight females between the ages of 18 and 34 were 30.5% and 30.3%, respectively [10]. The research by Cuong et al. [13] showed that the prevalence of underweight females in Ho Chi Minh City was 20.4%, and the survey from Hanoi in 2004 showed 22.4% underweight females between the ages of 25 and 34 [20].

The percentage of underweight females in the current study is higher compared to findings of previous studies. One possible explanation for this result may be related to factors influencing the development of disordered eating such as an increased exposure to Western culture, higher education levels, and socio economic development in Hanoi. Vietnamese students may focus more strongly on the body ideal from Western countries; for example, they might believe that body thinness is necessary to be successful in modern society. Another possible explanation could be that since 2008 the capital city Hanoi has grown two to three times because of the addition of the Hatay province. Therefore, the universities in Hanoi have more students coming from areas outside of the city whose family members are farmers with low incomes. The families have to pay tuition fees as well as room and board for their adult children when they go to the university in Hanoi. However, often their incomes are not sufficient to cover all the costs for the students living in Hanoi. This could be one of the reasons why some students become underweight since they can not afford a proper diet. This might also explain the results found in the surveys.

Previous studies conducted in Vietnam revealing the prevalence of underweight Vietnamese adults in urban areas as well as a growing prevalence of overweight individuals seem to indicate an important public health problem [13]. Unfortunately, there is very limited research addressing the prevalence and characteristics of an underweight Vietnamese population. Additionally, the BMI - criteria of the WHO are not specific enough to define what has to be considered as underweight in Asian people. Many Asian people tend to have a smaller body mass index compared to Western people and probably need a different set of criteria for the measure of body mass. The WHO has a recommendation for a BMI classification in Asians [17], but that only accounts for the differentiation between being overweight and obese.

A previous survey on participants in Ho Chi Minh City showed an increase in the number of overweight females. It found that 9.7% of females between the ages of 15 and 49 were overweight [21]. Additionally, the prevalence of overweight females between the ages of 20 and 29 in Ho Chi Minh City was 11.3% [13]. Another national survey showed that 2.5% of females between the ages of 18 and 34 were overweight [10]. A survey from Hanoi in 2004 showed that 18.1% of females between the ages 25 and 34 were overweight [20]. However, the result of the current study found only 1.5% as being overweight. Even if using the BMI- criteria for Asians, the percentages of overweight and obese females in the current study was 3.4% and 0.5%, respectively.

Hanoi and Ho Chi Minh City are the two largest cities in Vietnam and have populations of 6,936,900 and 7,818,200, respectively [22]. Cuong et al. [13] reported that being overweight is associated with higher economic status in Ho Chi Minh City. This was especially true for males between the ages of 40 and 49 [13]. The prevalence of overweight males in Vietnam might be attributed to being rich and successful, as weight increases with socio economic status and higher income, but these factors appear to be less relevant for women in Vietnam [13]. The fact that only young female university students participated in the current study could explain why the rate of being overweight was lower as compared to findings from previous studies.

### Disordered eating behaviors

48.8% of the participants reported two or more yes-responses on the SCOFF. According to Morgan et al. [15] this indicates that 48.8% of the Vietnamese participants have a very high possibility of having eating disorder symptoms. In the female group from a previous pilot study in Vietnamese high school students a similar percentage of 42.6% showed more than 2 yes-reponses [23]. One particularly interesting result is that 49.8% of the participants answered ‘yes’ to the SCOFF question number 2 (Do you worry that you have lost control over how much you eat?). This result is the same as the result in the previous pilot study [23], where most of the female high school students answered ‘yes’ to question number 2. The SCOFF question number 3 (Have you recently lost more than one stone in a 3-month period?) received the fewest ‘yes’ answers in both studies. The pilot study [23] and the current study only found a very low percentage of obese participants. The rate of overweight participants was 1.6% and 1.5%, respectively. These similar results from two Vietnam samples indicate it might be possible that despite a low body mass index many young females in Vietnam worry about losing control of their eating behaviors. This indicates that there might be a potential risk for young females in Vietnam of developing disordered eating behaviors.

However, the relationship between the SCOFF score and the EDI-2 scales showed a significant negative correlation. Furthermore, there was a slightly negative correlation between the SCOFF and the BMI. Regarding reliability, according to Schmitt [24], a Cronbach’s alpha of 0.60 or 0.70 indicates an acceptable reliability, and 0.70 or higher values indicate a good reliability. Cronbach’s alpha of the SCOFF showed an insufficient reliability of 0.28 in the current study. The SCOFF questionnaire has been validated in the US and in the UK and in these countries showed good validity and reliability as a screening instrument [18]. The SCOFF questionnaire has also been used as a screening test for children and adolescents in Germany, and it showed good validity as well [19]. In addition, the SCOFF in the French version showed good accuracy and reliability for the detection of women with EDs in the high-risk French student population [25]. In Asian cultures, the SCOFF score showed a positive relationship with the global score of the Eating Disorder Examination-Questionnaire and showed acceptable psychometric properties in Hong Kong secondary school students [26]. However, it has been suggested that translated versions need to be amended to address cultural issues [27]. Crosby et al. [27] mentioned that questions such as ‘do you make yourself sick’ may have different meanings outside of the UK. Furthermore, the Korean SCOFF questionnaires were inappropriate for improving the screening of eating disorders due to low internal consistency and reliability in the study by Jung et al. [28]. In summary, the SCOFF has shown inconsistent reliability in diverse cultural groups. The insufficient reliability and the lack of a significant relationship between the SCOFF and the EDI-2 in the current study also shows that there is a need for further studies using the SCOFF questionnaire in different samples and adapt it according to different cultural contexts.

Cronbach’s alpha of the EDI-2 indicated a good reliability with 0.85 in the current study. The subscales drive for thinness and body dissatisfaction of the EDI-2 showed a positive and significant relation to the BMI. In particular, the underweight group showed significantly lower scores on these two scales compared to normal weight and overweight individuals. This means that Vietnamese female students are dissatisfied with their body and feel a drive to be thin when they consider themselves to be overweight. There is no cut-off score for the EDI-2, but higher scores can be assumed to be related to a greater possibility of having disordered eating behaviors. Therefore, it is possible that the normal and overweight groups of young female Vietnamese have difficulty with drive for thinness or body dissatisfaction. As mentioned before, the ideal of thinness as seen through mass media products from Western cultures might be influencing this development in young female students in Vietnam.

Some further limitations of the current study need to be mentioned: The sample included only female students in the capital city in Vietnam. In addition, the translated questionnaires were self-report instruments and have not been validated in Vietnam yet. Furthermore, there is no information about dieting behavior or traditional eating behavior in Vietnam that can help to better understand the results.

## Conclusions

Despite the limitations, the current study shows a tendency in young female university students in urban Vietnam to become underweight and to develop disordered eating behaviors as well as a drive for thinness and body dissatisfaction according to the EDI-2. Additionally, findings of the SCOFF indicate that there is a risk group that shows signs of eating disorder symptoms. However, in order to verify this development more surveys and research are needed.

### Ethics consent

Ethics consent has been granted by the Human Research Ethics Committee of the Freiburg University Medical Center.
